# Dual versus single antiplatelet therapy in patients with nonminor ischemic stroke: a meta-analysis

**DOI:** 10.1055/s-0045-1802551

**Published:** 2025-02-24

**Authors:** Izabela Orlandi Môro, Gabriel Marinheiro, Marianna Leite, Gabriel de Almeida Monteiro, Agostinho C. Pinheiro, João Paulo Mota Telles

**Affiliations:** 1 Escola Superior de Ciências da Santa Casa de Misericórdia de Vitória, Vitória ES, Brazil.; 2 Universidade Federal do Ceará, Sobral CE, Brazil.; 3 Faculdade Santa Marcelina, São Paulo SP, Brazil.; 4 Harvard Medical School, Massachusetts General Hospital/Brigham and Women's Hospital, Department of Neurology, Boston MA, United States.; 5 Icahn School of Medicine at Mount Sinai, Elmhurst Hospital Center, Department of Internal Medicine, New York NY, United States.; 6 Universidade de São Paulo, Faculdade de Medicina, Departamento de Neurologia, São Paulo SP, Brazil.

**Keywords:** Ischemic Stroke, Dual Anti-platelet Therapy, Clopidogrel, Ticagrelor, Aspirin

## Abstract

**Background** Patients with ischemic stroke present a higher risk of stroke recurrence, neurological deterioration, and death. The benefit of dual antiplatelet therapy (DAPT) over single antiplatelet therapy (SAPT) among patients with minor ischemic stroke is well established; however, robust evidence is lacking for those with nonminor stroke.

**Objective** To describe the benefits and risks of DAPT versus SAPT in patients with nonminor ischemic stroke.

**Methods** We searched the PubMed, Embase, and Cochrane Library databases for articles published from inception to April 2024. Data were collected from randomized clinical trials and observational studies comparing DAPT to SAPT following nonminor ischemic stroke, defined by a score ≥ 4 on the National Institutes of Health Stroke Scale (NIHSS).

**Results** In total, 6 studies were included, comprising 12,480 patients. The NIHSS score at baseline from the selected studies ranged from 4 to 15. There was no significant difference between DAPT and SAPT for recurrent stroke (risk ratio [RR] = 0.91; 95% confidence interval [95%CI] = 0.82–1.01; *p* = 0.09; I ^2^ = 0%), ischemic stroke (RR = 0.89; 95%CI = 0.80–1.00; *p* = 0.05; I ^2^ = 0%) or hemorrhagic stroke (RR = 1.23; 95%CI = 0.41-3.99; *p* = 0.66; I ^2^ = 27%). Major bleeding was not significantly increased in the DAPT group compared with the SAPT group (RR = 0.87; 95%CI = 0.29–2.66; *p* = 0.81; I ^2^ = 44%). The overall analysis did not show a significant difference in all-cause mortality (RR = 0.72; 95%CI = 0.50–1.02; *p* = 0.07; I ^2^ = 0%).

**Conclusion** There was no difference between DAPT and SAPT regarding recurrent stroke, ischemic stroke, hemorrhagic stroke, major bleeding, or overall mortality.

## INTRODUCTION

Patients with ischemic stroke present a higher risk of experiencing stroke recurrence, as well as neurological deterioration and death. The prognosis for patients who had a subsequent stroke is often unfavorable, as the risk of disability and mortality increases. Stroke is still be one of the primary causes of death and disability worldwide. 1 2 3 4 Therefore, preventing the progression and recurrence of stroke is an important priority for patients who have suffered from an ischemic stroke. 5

The administration of single antiplatelet therapy (SAPT) with aspirin is routinely recommended after an acute ischemic stroke, within 24 to 48 hours after onset, based on its ability to reduce thrombotic complications. 3 In patients with minor, non-cardioembolic ischemic stroke (defined by a score ≤ 3 on the National Institutes of Health Stroke Scale 6 [NIHSS]) who did not receive IV alteplase, the benefit of dual antiplatelet therapy (DAPT) with clopidogrel plus aspirin within 24 hours of symptom onset is also well-established. Compared with aspirin monotherapy, the beneficial effect of aspirin plus clopidogrel was driven by a reduction in ischemic stroke. 5 6 7 8 According to the last revised guidelines of the American Heart Association (AHA) and American Stroke Association (ASA), 6 these treatments have strong and high-quality evidence. Recent meta-analyses 7 9 have also assessed the benefits of DAPT with ticagrelor plus aspirin in this context.

However, there is a lack of solid evidence supporting the use of DAPT among patients with nonminor ischemic stroke (NIHSS score ≥ 4), which leads to uncertainty regarding its value in this context. Such patients present more severe neurological impairment and larger areas of ischemic injury, which puts them at a higher risk of developing brain hemorrhage. 10 The use of DAPT may reduce early neurologic deterioration and improve the clinical outcomes of these patients if the risk of hemorrhage is controlled. 11 In light of this controversy, we have performed a systematic review and meta-analysis of the possible benefits and risks of DAPT with clopidogrel or ticagrelor plus aspirin versus SAPT in patients with nonminor acute ischemic stroke.

## METHODS

### Search strategy

We systematically searched the PubMed, Embase, and Cochrane Library databases from inception to April 2024 with the following search terms: *antiplatelet therapy* ; *dual antiplatelet* ; *clopidogrel* ; *ticagrelor* ; *dipyridamole* ; *aspirin* ; *ischemic stroke* ; *stroke* ; *brain infarct* ; *cerebral infarction* ; *mild* ; *moderate* ; *nonminor* ; and *non-minor* . The complete search strategy is detailed in Supplementary Material (only available online at https://www.arquivosdeneuropsiquiatria.org/wp-content/uploads/2024/11/ANP-2024.0198-Supplementary-Material.docx ; **Table S1** ). The references from all included studies, previous systematic reviews, and meta-analyses were also searched manually for any possible additional studies.

### Eligibility criteria

Two authors (IOM. and ML) independently screened titles and abstracts and fully evaluated the studies for eligibility. Discrepancies were settled by discussion with a third author (GM). The inclusion criteria were randomized controlled trials (RCTs) or observational studies comparing DAPT to SAPT, enrolling patients with nonminor ischemic stroke (defined by NIHSS score ≥ 4), and reporting at least one of the following outcomes: recurrent stroke, ischemic stroke, hemorrhagic stroke, major bleeding, or all-cause mortality.

The choice of DAPT with aspirin plus clopidogrel or ticagrelor was made based on a preliminary review of the literature which found guidelines 6 and meta-analyses 7 9 12 showing these are the most recognized antiplatelets used in DAPT. The exclusion criteria were studies that did not include a control group, studies with patients with minor stroke or transient ischemic attack, and studies in which the DAPT scheme used other antiplatelets instead of clopidogrel or ticagrelor with aspirin.

### Data extraction

Two authors (IM and GAM) conducted data extraction independently using the Microsoft Excel (Microsoft Corp., Redmond, WA, United States) software, following predefined search criteria and quality assessment. Disagreements were resolved through a consensus after discussing the reasons for the discrepancy with a third author (GM). The data extracted comprised the article's information (authors, publication year), type of the study (observational or RCT), population characteristics (study arm, number of patients, time window until antiplatelet therapy, follow-up time, NIHSS score range at baseline, sex, age, relevant medical history), intervention characteristics (antiplatelets used, doses and duration of treatment), and outcomes of interest (recurrent stroke, ischemic stroke, hemorrhagic stroke, all-cause mortality, and major bleeding). Subanalyses were performed restricted to RCTs, observational studies, and a selected population. Ethical approval was not required due to the nature of the study.

### Quality assessment

Nonrandomized studies were evaluated with the Risk of Bias in Non-Randomized Studies – of Interventions (ROBINS-I) tool. 13 In this scale, studies are scored as low, moderate, serious or critical risk of bias, in the domains of confounding, selection, interventions, missing data, measurement of outcomes, and reporting of results. Quality assessment of RCTs was performed using the Cochrane Risk of Bias 2 (RoB 2) tool, 14 in which studies are scored as high, low, or unclear risk of bias in five domains: selection, performance, detection, measurement, and reporting biases. Two independent authors (ML and GAM) completed the risk of bias assessment. Discrepancies were resolved through discussion with a third author (IM).

### Statistical analysis

This systematic review and meta-analysis followed the *Cochrane Handbook of Systematic Reviews of Interventions* and the Preferred Reporting Items for Systematic Reviews and Meta-analysis (PRISMA) statement guidelines. 15 16 The protocol was preregistered in the International Prospective Register of Systematic Reviews (PROSPERO; CRD42024536019).

We employed risk ratio (RR) with 95% confidence intervals (95%CIs) as the measure of effect size to report binary outcomes. There were no continuous outcomes. Heterogeneity was assessed with the Cochran Q test and I ^2^ statistics. Values of *p* < 0.10 and of I ^2^ ≥ 25% were considered significant for heterogeneity. We used the restricted maximum likelihood random-effects model. All statistical analyses were performed using the R software (R Foundation for Statistical Computing, Vienna, Austria), version 4.2.3.

## RESULTS

### Study selection and characteristics

**Figure 1:**
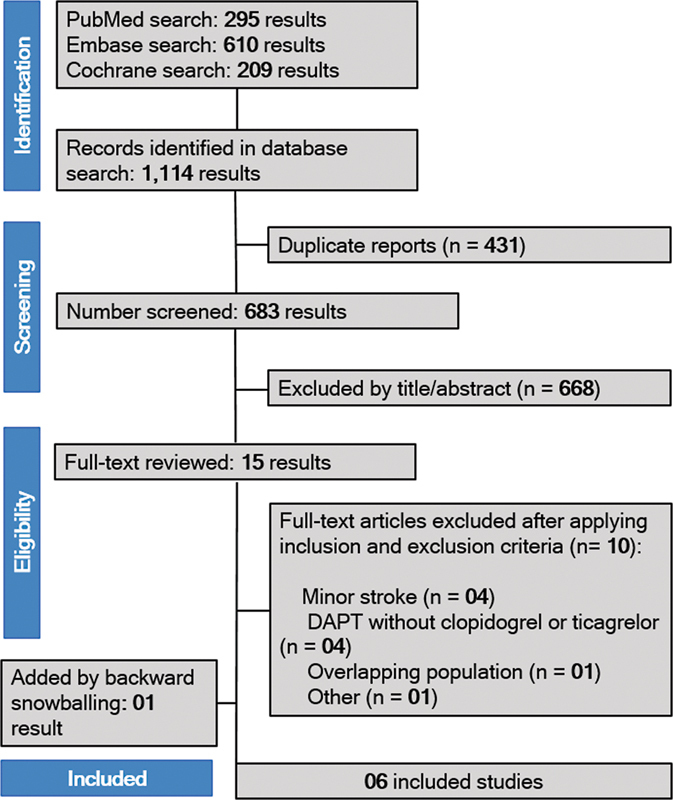
Preferred Reporting Items for Systematic Reviews and Meta-analysis (PRISMA) flow diagram of study screening and selection.

The initial search yielded 1,114 potential results. After the removal of duplicate records and ineligible studies, 15 articles remained and were thoroughly reviewed based on the inclusion criteria. Of these, five were included, and one was added by backward snowballing; therefore, the total sample comprised 12,480 patients from 2 RCTs and 4 observational studies. The study selection process is illustrated in Figure 1 using the PRISMA flow diagram.

Among all patients, 5,844 (46.8%) received DAPT, and 6,636 (53.2%) received SAPT. One study 10 included ticagrelor as part of the DAPT regimen, and the remaining studies 11 17 18 19 20 all used clopidogrel. Two studies 17 20 included clopidogrel or aspirin as a choice of SAPT, one 18 did not specify the SAPT regimen, and the remaining studies 10 11 19 used aspirin. The NIHSS score at admission ranged from 4 to 15. The time window until treatment ranged from 24 hours to 1 week. The follow-up ranged from 30 days to 1 year. The articles' information and population characteristics are reported in Table 1 , and the characteristics of the antiplatelet treatments are reported in Table 2 .

**Table 1:** Article data and population characteristics

Authors (year)	Type of study	Study arm (n)	Mean age (years)	Female sex (%)	NIHSS score: range at baseline	Hypertension: n (%)	Diabetes mellitus: n (%)	Previous ischemic stroke: n (%)	Smoker: n (%)	Dyslipidemia: n (%)
Chen et al. (2024; ATAMIS trial) 11	RCT	CLO + ASA (1,502)	65.7	35.3	4–10	931 (62)	401 (26.7)	482 (32.3)	503 (33.8)	19 (1.3)
		ASA (1,413)	66.0	34.7		879 (62.2)	341 (24.1)	457 (32.4)	464 (33.1)	19 (1.4)
Wang et al. (2024) 17	Non-RCT	CLO + ASA (781)	61.6	31.3	4–10	477 (61.1)	209 (26.8)	178 (22.8)	248 (31.8)	70 (9)
		SAPT ^§^ (1,633)	62.8	32.3		1.037 (63.5)	399 (24.4)	424 (26)	511 (31.3)	103 (6.3)
Downey et al. (2023) 20	Non-RCT	CLO + ASA (90)	66.9	48.9	≥ 4	76 (84.4)	33 (36.7)	NA	NA	NA
		SAPT ^§^ (68)	63.8	44		44 (64.7)	18 (26.5)	NA	NA	NA
Wang et al. (2021; THALEStrial) 10	RCT	TGL + ASA (1,671)	64.4	38.9	4–5	1.370 (82)	627 (37.5)	308 (18.4)	475 (28.4)	627 (37.5)
		ASA (1,641)	64.6	39.2		1.343 (81.8)	614 (37.4)	272 (16.6)	466 (28.4)	614 (37.4)
Khazaal et al. ^+^ (2021) 18	Non-RCT	CLO + ASA (148)	67.3	44.6	≥ 4	128 (86.5)	65 (43.9)	NA	42 (28.4)	93 (62.8)
		SAPT ^&^ (229)	66.4	53.5		168 (73.4)	75 (32.8)	NA	59 (25.8)	116 (50.7)
Kim et al. (2019) 19	Non-RCT	CLO + ASA (1652)	68.7	58.7	4–15	1126 (68.2)	576 (34.9)	301 (18.2)	564 (34.1)	489 (29.6)
		ASA (1652)	69.1	59.3		1121 (67.9)	574 (34.7)	293 (17.7)	569 (34.4)	486 (29.4)

Abbreviations: ATAMIS, Antiplatelet Therapy in Acute Mild-Moderate Ischemic Stroke ASA, acetylsalicylic acid (aspirin); CLO, clopidogrel; NA, not available; NIHSS, National Institutes of Health Stroke Scale; RCT, randomized controlled trial; SAPT, single antiplatelet therapy; TGL, ticagrelor; THALES, Acute Stroke or Transient Ischaemic Attack Treated with Ticagrelor and ASA for Prevention of Stroke and Death.

**Notes:**^+^ Data from the study by Khazaal et al. 18 refers to all patients, although we have only analyzed outcomes for those who did not receive tissue plasminogen activator (tPA) or thrombectomy; ^§^ refers to the use of aspirin or clopidogrel alone; ^&^ SAPT regimen not specified.

**Table 2:** Characteristics of the antiplatelet treatment

Authors (year)	Study arm (n)	Loading dose (mg)	Maintenance dose (mg)	Maintenance duration (days)	Time window for antiplatelet therapy	Duration of treatment (days)	Follow-up time (months)
Chen et al. (2024; ATAMIS trial) 11	CLO + ASA (1,502)	300 + 100	75 + 100 (day 2–14); 75 CLO or 100 ASA (day 15–90)	14/90	Within 48 hours	90*	3
	ASA (1,413)	100–300	100	90			
Wang et al. (2024) 17	CLO + ASA (781)	75 + 100	NA	30	Within 24 hours	90	12
	SAPT ^§^ (1,633)	75 CLO or 100 ASA	NA	NA			
Downey et al. (2023) 20	CLO + ASA (90)	NA	NA	NA	Within 1 week	NA	3
	SAPT ^§^ (68)	NA	NA	NA			
Wang et al. (2021; THALEStrial) 10	TGL + ASA (1,671)	180 + 300–325	90 + 75–100	30	Within 24 hours	30	1
	ASA (1,641)	300-325	75–100	30			
Khazaal et al. ^+^ (2021) 18	CLO + ASA (148)	600-300 + NA	75	NA	24 hours, median	5, mean	NA
	SAPT ^&^ (229)	NA	NA	NA			
Kim et al. (2019) 19	CLO + ASA (1,652)	NA	NA	NA	Within 24 hours	NA	3
	ASA (1,652)	NA	NA	NA			

Abbreviations: ASA, acetylsalicylic acid (aspirin); ATAMIS, Antiplatelet Therapy in Acute Mild-Moderate Ischemic Stroke; CLO, clopidogrel; NA, not available; SAPT, single antiplatelet therapy; TGL, ticagrelor; THALES, Acute Stroke or Transient Ischaemic Attack Treated with Ticagrelor and ASA for Prevention of Stroke and Death.

Notes: ^*^ The dual antiplatelet therapy (DAPT) group in the ATAMIS trial received CLO + ASA for 14 days. Then, the patients received CLO or ASA until day 90. ^+^ Data from the study by Khazaal et al. 18 refers to all patients, although we have only analyzed outcomes for those who did not receive tissue plasminogen activator (tPA) or thrombectomy. ^§^ Refers to the use of aspirin or clopidogrel alone. ^&^ SAPT regimen not specified.

### Pooled analysis of the included studies

#### *Efficacy outcomes*

**Figure 2:**
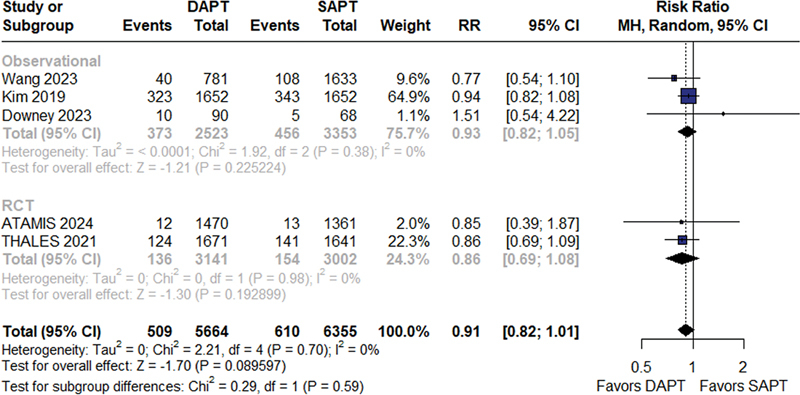
Recurrent stroke.

**Figure 3:**
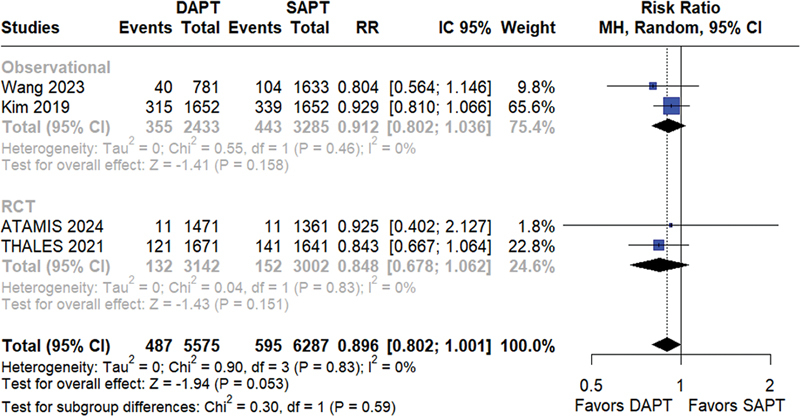
Ischemic stroke.

**Figure 4:**
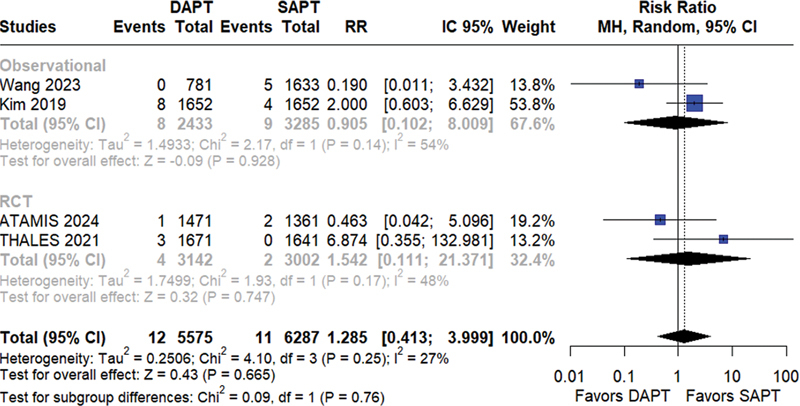
Hemorrhagic stroke.

There were no statistically significant differences in the rates of recurrent stroke between the DAPT and SAPT groups (RR = 0.91; 95%CI = 0.82–1.01; *p* = 0.09; I ^2^ = 0%; Figure 2 ). Neither were there statistically significant differences between the two groups regarding ischemic stroke (RR = 0.89; 95%CI = 0.80–1.00; *p* = 0.05; I ^2^ = 0%; Figure 3 ), and an analysis restricted to RCTs did not demonstrate a difference between these treatments (RR = 0.85; 95%CI = 0.68–1.06; *p* = 0.158; I ^2^ = 0%; Figure 3 ). Neither group showed benefits in terms of the reduction in the risk of hemorrhagic stroke (RR = 1.23; 95%CI = 0.41–3.99; *p* = 0.66; I ^2^ = 27%; Figure 4 ).

#### *Safety outcomes*

**Figure 5:**
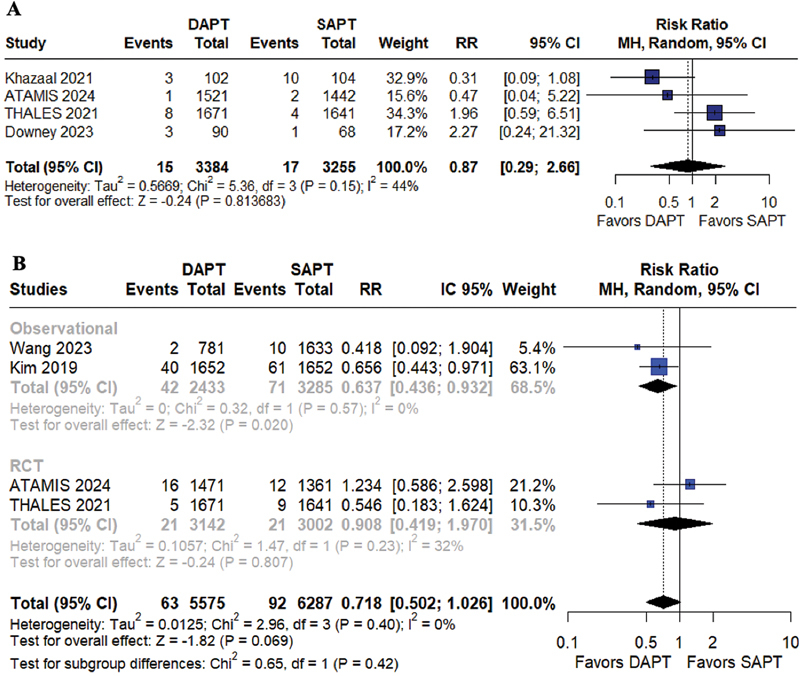
( **A** ) Major bleeding; ( **B** ) all-cause mortality.

Major bleeding was not significantly increased in the DAPT group compared with the SAPT group (RR = 0.87; 95%CI = 0.29–2.66; *p* = 0.81; I ^2^ = 44%; Figure 5A ). In the overall analysis, there was no difference in all-cause mortality between the groups (RR = 0.72; 95%CI = 0.50–1.02; *p* = 0.07; I ^2^ = 0%; Figure 5B ). Neithger were there differences in all-cause mortality in the analysis restricted to RCTs (RR = 0.91; 95%CI = 0.42–1.97; *p* = 0.81; I ^2^ = 32%; Figure 5B ), although, when restricted to observational studies, we found benefits on the DAPT group in terms of a reduction in all-cause mortality (RR = 0.63; 95%CI = 0.44–0.93; *p* = 0.02; I ^2^ = 0% Figure 5B ).

#### *Subanalysis in a selected population*

A subanalysis was conducted excluding data from the Acute Stroke or Transient Ischaemic Attack Treated with Ticagrelor and ASA for Prevention of Stroke and Death (THALES) 10 trial. We did this because this trial contained patients with the lowest NIHSS score range at baseline (4–5) among the included studies. We aimed to assess if we would find a difference in the efficacy and safety of DAPT compared to SAPT only in the patients of studies with the highest NIHSS score ranges, since there is no consensus in the literature about the definition of nonminor stroke. 21

There were no significant differences in terms of stroke recurrence between the groups (RR = 0.92; 95%CI = 0.82–1.05; *p* = 0.20; I ^2^ = 0%; **Supplementary Material Figure S3A** : online only), nor regarding ischemic stroke (RR = 0.91; 95%CI = 0.80–1.04, *p* = 0.15; I ^2^ = 0%; **Supplementary Material Figure S3B** : online only). Neither were there significant differences between the groups regarding hemorrhagic stroke (RR = 0.87; 95%CI = 0.21-3.68; *p* = 0.84; I ^2^ = 30%; **Supplementary Material Figure S3C** : online only), major bleeding (RR = 0.54; 95%CI = 0.17–1.73; *p* = 0.29; I ^2^ = 14%; **Supplementary Material Figure S3D** : online only), nor all-cause mortality (RR = 0.76; 95%CI = 0.74–1.23; *p* = 0.26; I ^2^ = 26%; **Supplementary Material Figure S3E** : online only).

### Risk of bias assessment

The individual assessment of each study included is presented in **Supplementary Material Figures S1** and **S2** (online only). Overall, most of the included studies presented a low risk of bias, since the randomized studies provided reliable protocols and blinding procedures. Some observational studies presented a moderate risk of bias because of possible confounding factors, but this was not deemed critical for the quality of the pooled meta-analysis due to the performance of multivariate analysis. Only one cohort study 17 was also considered to present a moderate risk of bias because of deviations from one study arm to another close to the time of follow-up. Another study 19 was considered to present an overall low risk of bias due to propensity score matching.

## DISCUSSION

The present systematic review and meta-analysis indicated that there were no significant differences between the DAPT and SAPT regimens for patients with nonminor stroke in terms of stroke recurrence, ischemic stroke, hemorrhagic stroke, major bleeding, and all-cause mortality. Statistical significance was only found in the analysis restricted to observational studies regarding the superiority of DAPT in reducing all-cause mortality, which was not confirmed in the RCT-only or all-study analyses.

The use of DAPT in the context of minor or mild-to-moderate ischemic stroke and transient ischemic attack (TIA) was the focus of previous important and recognized randomized clinical trials. 22 23 24 25 These trials evaluated ischemic stroke patients with NIHSS scores ≤ 3 or ≤ 5, or TIA, and found superiority of DAPT over SAPT in reducing the risk of stroke. Moreover, meta-analyses 7 9 have been endorsed these results. In consequence, the evidence for the use DAPT in this context is strong and recommended in important guidelines. 5 6

On the contrary, to date, only a few studies have assessed the efficacy and safety of DAPT among patients with higher NIHSS scores, that is, nonminor ischemic stroke defined by an NIHSS score ≥ 4. The Antiplatelet Therapy in Acute Mild-Moderate Ischemic Stroke (ATAMIS) 11 trial was the first large-scale, multicenter, RCT in this context. Also, recently, a subanalysis 10 of the THALES 24 trial comprised the patients NIHSS scores between 4 and 5. The THALES 24 trial originally included patients with NIHSS ≤ 5, and, posteriorly, the authors considered the analysis of the group with NIHSS scores from 4 to 5 was worth performing because it could result in outcomes different than those of the patients with a minor stroke defined by NIHSS score ≤ 3. Since to date only these 2 RCTs have been conducted on patients with NIHSS score ≥ 4, the observational studies 17 18 19 20 also provide essential data that helps analyze this matter. The patients in one of the observational studies 19 included in our meta-analysis, pertinently, are those with nonminor stroke who did not meet the criteria for inclusion in the Clopidogrel in High-risk Patients with Acute Non-Disabling Cerebrovascular Events (CHANCE) 22 or Platelet-Oriented Inhibition in New TIA and Minor Ischemic Stroke (POINT) 23 trials, since these trials evaluated patients with NIHSS scores ≤ 3.

There is no consensus among international stroke organizations on the definition of minor stroke. One study 26 compared the different definitions used in the literature and found that ‘conscious patients scoring ≤ 1 on every NIHSS item’ and ‘patients with NIHSS ≤ 3’ presented the best short- and medium-term outcomes, so we can assume that minor stroke patients, by these definitions, are those who have the best chance of achieving a positive outcome. In this sense, patients with nonminor stroke may respond differently to DAPT than those with minor stroke, due to more severe neurological impairment and larger areas of ischemic injury, which may increase their risk of hemorrhage. 10 Therefore, DAPT could benefit patients with nonminor stroke if their bleeding risk can be controlled. In light of this, in the present systematic review and meta-analysis of 6 studies comprising 12,480 patients, we have analyzed DAPT versus SAPT after a nonminor ischemic stroke, defined by NIHSS score ≥ 4.

Stroke outcomes range drastically, from no disability to death, which can be influenced by the patient's premorbid status, stroke severity, acute treatment, or environment. 27 Our findings demonstrate that the prevalence of well-known risk factors, such as hypertension, diabetes mellitus, and smoking, was similar between the studies analyzed and the DAPT and SAPT groups. One interest baseline characteristic that varied impressively among the studies was the prevalence of dyslipidemia, which ranged from 1.3% to 62.8%. The lowest prevalence was found in the ATAMIS 11 trial, which included patients from hospitals in China. The highest prevalence was found in the study by Khazaal et al., 18 which included patients from hospitals in the United States. These findings are consistent with those of a meta-analysis 28 that also found lower rates of dyslipidemia among the Chinese population. Specifically, the authors found a prevalence of 9% of dyslipidemia among the Chinese population and of 30% among the population of Caucasians who had had an ischemic stroke. This points to important cultural differences that may require different care in stroke prevention among diverse populations.

Concerning the occurrence of bleeding events, the current study found that the treatment groups did not differ overall. This outcome is of particular relevance, because one of the main concerns about DAPT therapy is the increased risk of bleeding, 29 30 which has important prognostic implications, since it can influence long-term functional outcomes. 31 Khazaal et al. 18 were the only ones to include patients who received tissue plasminogen activator (tPA) or thrombectomy. These treatments may lead to bleeding complications, 32 33 and it can be confusing if the antiplatelet therapy was the real reason for the bleeding. Therefore, we analyzed only the subgroup that did not receive these treatments. However, the authors ^18^ pointed out that it is still reassuring that intracranial hemorrhage in the DAPT group was very low in their study.

It is also important to highlight that one outcome of extreme importance is neurologic deterioration, since patients with nonminor ischemic stroke may be more susceptible to it, and there is an association between neurologic deterioration and poor outcomes after nonminor ischemic stroke. 10 34 Unfortunately, only the ATAMIS 11 trial evaluated this outcome, and we cannot provide a meta-analysis for it. Besides that, it is worth reporting that the ATAMIS 11 trial found a significant difference in the risk of early neurologic deterioration between the DAPT and SAPT groups, indicating that DAPT improves early neurologic function in nonminor ischemic stroke. We underline the need for more trials evaluating that outcome.

The time window for antiplatelet therapy is extremely important to achieve the best outcomes. In the present meta-analysis, 4 studies 10 17 18 19 used the 24-hour time window, 1 study, 11 the 48-hour, and another one 20 analyzed patients in whom DAPT was initiated within 1 week, which may have contributed to different outcomes. The duration of antiplatelet treatments also varied among the included studies, ranging from 5 to 90 days, or it was not not reported, which can affect the risk of hemorrhagic stroke and major bleeding, since antiplatelet agents increase bleeding risk the longer they are used. The European Stroke Organization 5 makes a strong recommendation for the use of 21 days of DAPT within 24 hours for patients with minor stroke (NIHSS score ≤ 3), and a weak recommendation for 30 days of DAPT in mild-to-moderate ischemic stroke (NIHSS score ≤ 5) within 24 hours. In turn, the last revised guidelines from the AHA and ASA 6 recommend DAPT for minor stroke (NIHSS score ≤ 3) starting within 24 hours of symptom onset and continuing for 21 days. These recommendations differ in terms of the time for initiation and the duration of DAPT in some of the studies included in the present review, and it may have partly influenced the outcomes found.

Furthermore, we found that DAPT was statistically significantly superior to SAPT in reducing all-cause mortality only in an analysis restricted to the observational studies. Although caution is needed, this result indicates that it may be suitable to choose DAPT in the context of nonminor ischemic stroke, which is an encouragement to conduct new clinical trials. Perhaps a selection of patients with lower baseline hemorrhage risk might show that more patients could benefit from dual therapy, particularly in the first days.

New evidence is emerging on different treatment approaches for nonminor ischemic stroke, such as the use of DAPT. This is extremely necessary, since patients with nonminor ischemic stroke are at a higher risk of deteriorating and of poor outcomes when compared to those with low-severity stroke, 21 and this directly influences the patient's quality of life and increases social health costs. 35 36

## Limitations

The current study has several limitations that need to be addressed. Firstly, to date, only a few studies have evaluated the use of DAPT in patients with nonminor stroke, so we had to include observational studies to gather more data on this topic. We must remember that observational studies, although they provide valuable insights, are not the most robust source of evidence. Furthermore, the studies vary in terms of the time window for initiating antiplatelet therapy, duration of treatment, and follow-up time.

In conclusion, the present research highlights and discusses important topics regarding the use of DAPT in patients with nonminor ischemic stroke, providing clinicians and researchers with a broad view of this topic. Our findings show that there was no difference between DAPT and SAPT in terms of recurrent stroke, ischemic stroke, hemorrhagic stroke, major bleeding, nor overall mortality. Further RCTs need to be conducted so that the evidence can gain strength in this relevant clinical subject.
